# Lead‐Free Halide Perovskites for Direct X‐Ray Detectors

**DOI:** 10.1002/advs.202300256

**Published:** 2023-05-26

**Authors:** Xiangshun Geng, Yu‐Ang Chen, Yuan‐Yuan Li, Jun Ren, Guan‐Hua Dun, Ken Qin, Zhu Lin, Jiali Peng, He Tian, Yi Yang, Dan Xie, Tian‐Ling Ren

**Affiliations:** ^1^ School of Integrated Circuit & Beijing National Research Center for Information Science and Technology (BNRist) Tsinghua University Beijing 100084 P. R. China; ^2^ Beijing National Research Center for Information Science and Technology Tsinghua University Beijing 100084 P. R. China

**Keywords:** fabrication methods, lead‐free perovskites, radiation detection, X‐ray detectors

## Abstract

Lead halide perovskites have made remarkable progress in the field of radiation detection owing to the excellent and unique optoelectronic properties. However, the instability and the toxicity of lead‐based perovskites have greatly hindered its practical applications. Alternatively, lead‐free perovskites with high stability and environmental friendliness thus have fascinated significant research attention for direct X‐ray detection. In this review, the current research progress of X‐ray detectors based on lead‐free halide perovskites is focused. First, the synthesis methods of lead‐free perovskites including single crystals and films are discussed. In addition, the properties of these materials and the detectors, which can provide a better understanding and designing satisfactory devices are also presented. Finally, the challenge and outlook for developing high‐performance lead‐free perovskite X‐ray detectors are also provided.

## Graphical abstract

Lead‐free perovskites have fascinated significant research attention for direct X‐ray detection. This review describes the current research progress in the synthesis methods of lead‐free perovskites, fundamental principles, and development of applications of X‐ray detectors. In addition, the challenge and outlook for developing high‐performance lead‐free perovskite X‐ray detectors are also discussed.

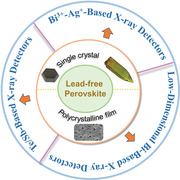

## 1 Introduction

Radiation detectors are widely applied in medical imaging, pulsar navigation, and security inspection by converting captured X/*γ*‐ray into electrical signals.^[^ ^1^, ^2^, ^3^, ^4^, ^5^, ^6^, ^7^, ^8^ ^]^ There are two available strategies for radiation detection: direct conversion of high energy photons into electrical signals in detectors and indirect conversion into visible light in scintillators.^[^ ^9^, ^10^, ^11^, ^12^ ^]^ Traditional scintillators, such as CsI (CsI:TI) and NaI (NaI:TI), possess high‐light yield and high‐energy resolution and have been successfully applied in commercialization. However, these indirect X‐ray detectors inevitably cause light scattering and radioluminescence afterglow, which compromises the spatial resolution of the output dynamic images.^[^ ^13^, ^14^, ^15^ ^]^ In contrast, the direct X‐ray detectors with a straightforward device configuration can minimize harmful scattering effects and simplify the conversion process to obtain higher resolution and dynamic imaging effects, lending it a significant competitive edge.^[^ ^16^, ^17^, ^18^, ^19^, ^20^ ^]^ Consequently, there is a surge of interest in developing cost‐effective direct X‐ray detectors. Crystalline Si, *α*‐Se, and CdTe are the representative of conventional radiation detection materials commonly used for direct conversion detectors, but these materials still suffer from their own drawbacks. For instance, Si and *α*‐Se have poor stopping capacity for high‐energy rays and CdTe presents the uniformity of the charge transport as well as complicated preparation processes, which are major challenges in practice.^[^ ^21^, ^22^, ^23^ ^]^

Perovskites, as a superstar material, have demonstrated excellent optoelectronic performance because of their strong radiation absorption ability (*α*∝*Z* ^4^/*AE* ^3^, where *Z* is the average atomic number, *A* represents the atomic mass, and *E* represents the X‐ray photon energy), large mobility‐lifetime (*µτ*) products, high defect tolerance, long carrier diffusion length, and facial fabrication processes.^[^ ^24^, ^25^, ^26^, ^27^, ^28^ ^]^ High‐performance detectors which apply perovskite materials have emerged as powerful candidates for next‐generation radiation devices. Lead (Pb) containing perovskites have a high average atomic number and excellent X‐ray absorption ability compared to that of Pb‐free perovskites. Additionally, the superior properties of Pb‐based perovskite X‐ray detectors are also originated from small exciton binding energy, high symmetry, high electronic dimensionality, and the unique atomic electronic configuration of Pb (the lone‐pair Pb 6s, Pb 6p orbitals contribute the conduction band minimum and the strong spin–orbit coupling), which results in high *µτ* products and high collection efficiency of photogenerated carriers. Although the vast majority of high‐performance X‐ray detectors are based on Pb‐containing perovskites, the toxicity of Pb potentially prevents their commercialization process. Therefore, lead‐free perovskites have emerged as the most promising alternative because of its nontoxicity and made extraordinary progress by using nontoxic elements, such as Bi and Sn, to replace Pb.^[^ ^29^, ^30^, ^31^, ^32^, ^33^ ^]^ To date, lead‐free perovskites in various forms including polycrystalline films and single crystals have been recently used in the fabrication of direct radiation detectors. Most of the reported lead‐free perovskite detectors have better sensitivity and the lower detection limit than that of ɑ‐Se (20 µC Gy_air_^−1^ cm^−2^ and 5500 nGy_air_ s^−1^).^[^ ^34^, ^35^, ^36^ ^]^ For example, Dun et al. fabricated the pixeled Cs_2_AgBiBr_6_ perovskite array X‐ray detectors, which exhibit a high X‐ray sensitivity of ≈1.91 × 10^4^ µC Gy_air_^−1^ cm^−2^.^[^ ^37^ ^]^ By eliminating inclusions of CsBr‐rich phases and restraining the trap‐state density, the detector based on Cs_3_Bi_2_Br_9_ single crystal has an ultralow detection limit of 0.58 nGy_air_ s^−1^ for hard X‐ray imaging.^[^ ^38^ ^]^ Moreover, researchers have revealed the effects of materials and device structures on the key figures‐of‐merit of the detectors, which could provide valuable insights into the device response mechanism.^[^ ^39^, ^40^, ^41^ ^]^ Therefore, it is of great significance to systematically summarize some cutting‐edge research and comb out the guidelines for designing advanced radiation detectors. We find that many reviews focus on the perovskite indirect X‐ray detectors, perovskite single‐crystal X‐ray detectors, and lead halide perovskites X‐ray detectors, et al.,^[^ ^42^, ^43^, ^44^ ^]^ but there is a lack of systematic reviews on lead‐free perovskite X‐ray detectors.

Here, we aim at summarizing the latest progress of lead‐free perovskite as a direct semiconductor for X‐ray radiation detectors. This review starts from the synthesis of the promising single crystal and polycrystalline film lead‐free perovskites, for which we clearly demonstrate all the current synthesis strategies of perovskite materials. Next, we briefly explain the detection mechanism and the main performance parameters of the detectors. Then, we give a critical review of the recent progress of lead‐free perovskite X‐ray detectors and classify these works as Bi^3+^‐Ag^+^, low‐dimensional Bi‐based, and Te/Sb‐based perovskites detectors and analyze the influence of material characteristics on device performance. Finally, the main challenges faced by lead‐free perovskite materials and X‐ray detectors are presented alongside the prospects and possible solutions that may allow for the achievement of commercial, stable, and high‐performance radiation devices.

## 2 Fabrication Methods

**Figure 1:**
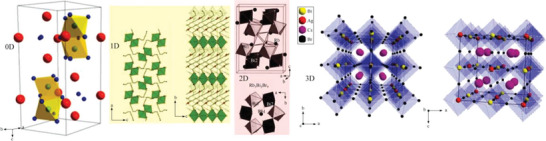
Schematic illustration of molecular 0D, 1D, 2D, and 3D perovskites. Reproduced with permission.^[^ ^45^ ^]^ Copyright 2020, WILEY‐VCH Verlag GmbH & Co. KGaA.

Lead‐free perovskites have experienced rapid development and exhibit rich structural diversity. The structural dimension of perovskite at the molecular level can be divided into 0D, 1D, 2D, and 3D, as shown in **Figure** **1**.^[^ ^45^, ^46^, ^47^ ^]^ The 3D Bi‐based perovskite are also known as double perovskites, with a general chemical formula of A_2_B′B″X_6_, in which A is cation, B^′^ is monovalent cation, B^″^ is trivalent cation, and X is halogens (Cl^−^, Br^−^, I^−^). Reducing the dimensionality makes perovskite a 2D structure such as Rb_3_Bi_2_I_9_ perovskite. In this structure, these bridged halides are placed in the AX_3_ layer together with Bi, and all bridged atoms are shared with the other three octahedra to form a corrugated Bi_2_X_9_ layer. Besides, the 2D double perovskites, such as (BA)_2_CsAgBiBr_7_, (DFPIP)_4_AgBiI_8_, and (I‐C_4_H_8_NH_3_)_4_AgBiI_8_ can be obtained by organic cations A‐site alloying or substitution. In those materials, the two kinds of the octahedron (AgX_6_ and BiX_6_) are arranged alternately to form an inorganic layer. The bilayers of organic cations are embedded between the inorganic layers and are connected. Further dimensional reduction produces 1D structure perovskites (such as zigzag (H_2_AETH)BiI_5_). The 0D structure perovskites (such as MA_3_Bi_2_I_9_) have a face shared binuclear octahedra, which forms a [Bi_2_X_9_]^3−^ complex where the A cation fills the voids at the terminal sides and blocks any further connections. For different dimensions, single crystal and polycrystal lead‐free perovskite films have diverse preparation methods.

### 2.1 Single Crystals

Low‐temperature solution crystallizations are a common method for the synthesis of single‐crystal lead‐free perovskites. This method has the advantages of simple equipment and process, good crystallinity, and easy preparation. At present, the reported growth strategies for perovskite crystals include temperature gradient methods, antisolvent crystallization methods, liquid diffused separation crystallization (LDSC), and solvent volatilization methods.^[^ ^48^, ^49^, ^50^, ^51^, ^52^ ^]^ With the development of synthesis technologies, the Bridgman method has also been used to grow lead‐free single‐crystal perovskites.^[^ ^53^, ^54^ ^]^ These growth routes for single‐crystal perovskite in X‐ray detectors have realized great progress. In this section, the crystal growth methods and corresponding basic principles of lead‐free single‐crystal perovskite are comprehensively summarized and discussed.

#### 2.1.1 Temperature Gradient Method

Heating‐up crystallization and cooling‐down crystallization are important strategies for preparing crystals by temperature gradient method. The solubility of perovskite increases or decreases regularly with the temperature change. The characteristic of solubility with temperature makes it possible to prepare crystals by the temperature gradient method.

**Figure 2:**
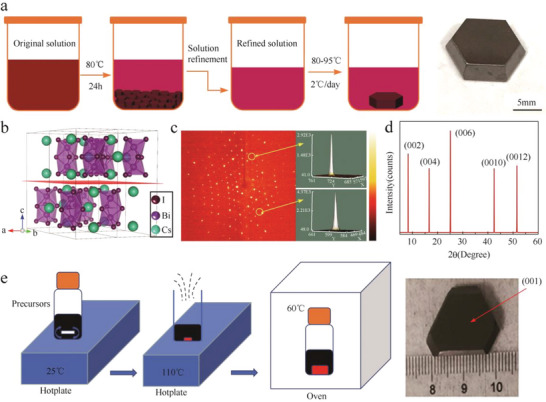
a) Sketch of the heating‐up crystallization set‐up for Cs_3_Bi_2_I_9_ single‐crystal perovskite growth. b) Crystal structure of Cs_3_Bi_2_I_9_ perovskite. c) Single‐crystal X‐ray diffraction spots of Cs_3_Bi_2_I_9_ single crystal. d) XRD of the (002) facet of the Cs_3_Bi_2_I_9_ single crystal. Reproduced with permission.^[^ ^55^ ^]^ Copyright 2020, Springer Nature. e) Preparation of (NH_4_)_3_Bi_2_I_9_ single‐crystal perovskite by cooling‐down crystallization. Reproduced with permission.^[^ ^57^ ^]^ Copyright 2019, Springer Nature.

Heating‐up crystallization is suitable for the situation in which the solubility of perovskite decreases with increasing temperature. In this growth method, the perovskite precursors are usually dissolved in the polar organic solvent. Zhang et al. obtained a large Cs_3_Bi_2_I_9_ single crystal (12 mm × 12 mm × 3 mm) using this method.^[^ ^55^ ^]^ They dissolved CsI and BiI_3_ in a mixed solvent (dimethylformamide (DMF)/dimethyl sulfoxide = 7:3) to prepare perovskite precursor. After careful filtration, the solution is placed into a temperature‐controlled oven at 80 °C. When a large number of crystals participate at the bottom of the container, the upper portion of the refined solution is then transferred into another container to grow large crystals. And then, the refinement solution was heated with the ramp rate of 2 °C day^−1^ from 80 to 95 °C. **Figure** **2**a is the crystal growth process and photograph of Cs_3_Bi_2_I_9_ perovskite single crystals with a well‐defined octahedron. The crystal structure in Figure 2b demonstrates different dimer [Bi_2_I_9_]^3−^ anions along the ab (00l) plane layered arrangement. The well‐aligned lattice diffraction spots and the (001) diffraction peak demonstrate high crystalline quality (Figure 2c,d).

The solubility of perovskite in the acidic solution such as HX (X = Cl, Br, I) increases with increasing temperature. Based on this principle, perovskite crystals can be prepared by the cooling‐down crystallization method. There are three regions in the single‐crystal growth model, including nucleation zone, growth zone, and dissolution zone, separated by the supersolubility and solubility curves. The key strategy for effective control of solubility and supersolubility curves is to avoid the HX volatilization. In the dissolution zone, all the raw materials are dissolved by heating the solution to a higher temperature. The saturated solution requires cooling to a temperature slightly above the supersolubility curves to enter the nucleation zone, and nuclei will spontaneously form. Subsequently, the solution was kept in the growth zone to grow larger crystals. In 2019, Yin et al. employed solubility and supersolubility as quantitative indicators to grow Cs_2_AgBiBr_6_ single crystal with high resistivity of 3.31 × 10^10^ Ω cm.^[^ ^56^ ^]^ Meanwhile, Zhuang et al. added bismuth oxide (Bi_2_O_3_) and ammonia iodide (NH_4_I) into hydroiodic acid (HI) and fully dissolved under magnetic stirring at room temperature.^[^ ^57^ ^]^ The obtained solution was concentrated on a hot plate at 110 °C and then quickly placed in a thermostat at 60 °C for crystal growth. It should be noted that crystal growth should not be interrupted to avoid massive nucleation (Figure 2e). The (NH_4_)_3_Bi_2_I_9_ single‐crystal perovskite with dimensions of 21 × 20 × 7 mm^3^ after 5 days of growth was obtained. In addition, the cooling‐down crystallization method also permits rapid growth of other lead‐free perovskites, such as MA_3_Sb_2_I_9_, Cs_2_AgInCl_6_, and Cs_2_AgBiBr_6_ single crystals.^[^ ^58^, ^59^, ^60^ ^]^

#### 2.1.2 Antisolvent Crystallization Methods

**Figure 3:**
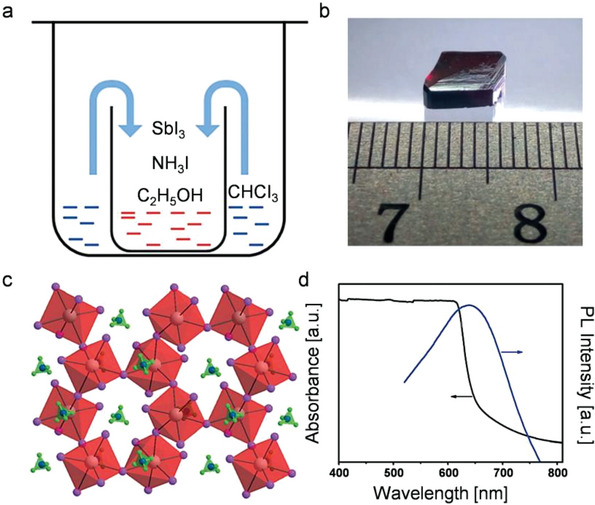
a) Schematic diagram of the antisolvent crystallization process for growing perovskite single crystals. b) The photograph of as‐prepared (NH_4_)_3_Sb_2_I_9_ single crystals. c) Crystal structure of (NH_4_)_3_Sb_2_I_9_ perovskite. d) The absorbance and the PL spectrum of (NH_4_)_3_Sb_2_I_9_ perovskite. Reproduced with permission.^[^ ^62^ ^]^ Copyright 2017, Wiley‐VCH Verlag GmbH &Co. KGaA.

One of the most typical methods to crystallize a material is antisolvent precipitation. Supersaturation can be achieved simply by exposing the perovskite solution to another less polar solvent (or multiple ones) in order to allow the perovskite to start nucleating. The antisolvent crystallization method was employed by Shi et al. to grow the first reported CH_3_NH_3_PbX_3_ (X = Br, I) single‐crystal perovskite.^[^ ^61^ ^]^ According to this facile method, the authors obtained (NH_4_)_3_Sb_2_I_9_ single crystals with side lengths of around 5.7 mm and thicknesses of up to 2.1 mm.^[^ ^62^ ^]^ In addition, the hole mobility (4.8 cm^2^ V^−1^ s^−1^) and electron mobility (12.3 cm^2^ V^−1^ s^−1^) are comparable to that of 0D MA_3_Bi_2_I_9_ single crystal prepared by seed‐crystal‐assisted constant‐temperature evaporation method.^[^ ^63^ ^]^ This method provides a simple strategy for synthesizing bulk single crystals with potential high‐performance X‐ray detection. The schematic diagram of the experiment is shown in **Figure** **3**a. NH_4_I and SbI_3_ are added to the anhydrous ethanol and the precursor is fully dissolved under magnetic stirring. Chloroform (CHCl_3_), which is used as the antisolvent, is added to the beaker. Under the condition that the beaker is sealed, CHCl_3_ will spontaneously diffuse into the solution and form large‐size (NH_4_)_3_Sb_2_I_9_ single crystals after a few days (Figure 3b). The single crystal presents a layered structure, in which the six I atoms form an octahedron with Sb at the center and an N atom at the center of a tetrahedron consisting of four H atoms (Figure 3c). Figure 3d exhibits an absorption onset at 645 nm and the photoluminescence (PL) spectrum presents a peak at 639 nm. In addition, the space‐confined antisolvent‐assisted crystallization method can achieve the growth of lead‐free perovskite with a specific thickness. For example, Dong et al. dripped the filtered Cs_3_Bi_2_I_9_ precursor solution onto octadecyltrichlorosilane‐treated glass and clamped it with another UV ozone‐treated indium tin oxide (ITO) or ITO/SnO_2_ substrate, and placed it in a sealed bottle filled with toluene. The Cs_3_Bi_2_I_9_ perovskite single crystals with a thickness of ≈1 µm are obtained on ITO or ITO/SnO_2_ substrate after 3–5 days of growth at 60 °C.^[^ ^64^ ^]^

#### 2.1.3 Bridgman Technique

**Figure 4:**
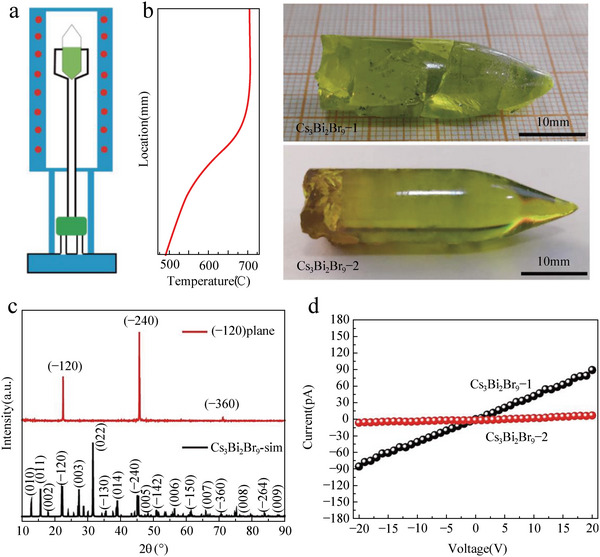
a) The basic structure of the vertical Bridgman furnace and optimized temperature field for crystal growth. b) Photographs of the as‐grown single crystal Cs_3_Bi_2_Br_9_‐1 (upper) and optimized growth of Cs_3_Bi_2_Br_9_ SC‐2 (lower). c) XRD patterns of (−120) plane. d) *I*–*V* curves of Cs_3_Bi_2_Br_9_ SC‐1 and Cs_3_Bi_2_Br_9_ SC‐2. Reproduced with permission.^[^ ^70^ ^]^ Copyright 2021, Science China Press and Springer‐Verlag GmbH Germany, part of Springer Nature.

The Bridgman technique was proposed by Bridgman in 1925.^[^ ^65^ ^]^ It is the most efficient way to grow certain semiconductor and metal crystal ingots or boules. This technique involves heating the polycrystalline materials in a container to melt them, and then slowly cooling them from the end where the seed crystal is located. Different kinds of perovskite, such as CsSnI_3_, CsCu_2_I_3_, and CsPbX_3_ (X = Cl, Br, I), have been grown by the Bridgman method.^[^ ^66^, ^67^, ^68^ ^]^ However, this high‐cost method will inevitably increase the production cost of X‐ray detectors. **Figure** **4**a demonstrates the basic structure of the Bridgman furnace. The single‐crystal materials grow gradually along the length of the container. This process can be carried out in vertical or horizontal geometry, which can be divided into the vertical Bridgman method and the horizontal Bridgman method. In 2017, McCall et al. prepared single crystals of the wide‐bandgap halide perovskites A_3_M_2_I_9_ (A = Cs, Rb; M = Bi, Sb) by the Bridgman method and investigated its optical and electronic properties.^[^ ^69^ ^]^ Afterward, halide perovskite crack‐free Cs_3_Bi_2_Br_9_ single crystals (Cs_3_Bi_2_Br_9_ SC‐2) with a diameter of 12 mm and length of ≈40 mm are successfully grown by using a modified Bridgman method for the first time (Figure 4b).^[^ ^70^ ^]^ The resistivity and transmittance of yellow transparent Cs_3_Bi_2_Br_9_ single crystal are ≈6.8 × 10^11^ Ω cm and ≈80%, respectively. The carrier mobility of the (−120) plane is 0.17 cm^2^ V^−1^ s^−1^, and the trap density is 9.7×10^10^ cm^−3^. In crystal growth, growth rate, cooling rate, and temperature gradient have a great influence on crystal quality. When a faster growth rate (0.5 mm h^−1^) and cooling rate (4 °C h^−1^) are adopted, cracks appear in the grown Cs_3_Bi_2_Br_9_ SC‐1 crystals. Figure 4c shows three diffraction peaks of the finely polished Cs_3_Bi_2_Br_9_ SC plates present at the (−120) diffraction plane. The Cs_3_Bi_2_Br_9_ SC‐2 exhibits a higher resistivity for the (−120) plane compared with that of Cs_3_Bi_2_Br_9_ SC‐1 (Figure 4d). In the following year, Li et al. further improved the crystal quality by eliminating inclusions of CsBr‐rich phases and reducing the grain boundaries and voids, leading to an enhanced resistivity of 1.41 × 10^12^ Ω cm, low trap density of 9.96 × 10^8^ cm^−3^, and a mobility lifetime product of 8.32 × 10^−4^ cm^2^ V^−1^.^[^ ^38^ ^]^ Interestingly, the optimized crystal quality improves the sensitivity of the X‐ray detector from ≈230.4 to 1705 µC Gy_air_^−1^ cm^−2^.

#### 2.1.4 LDSC

**Figure 5:**
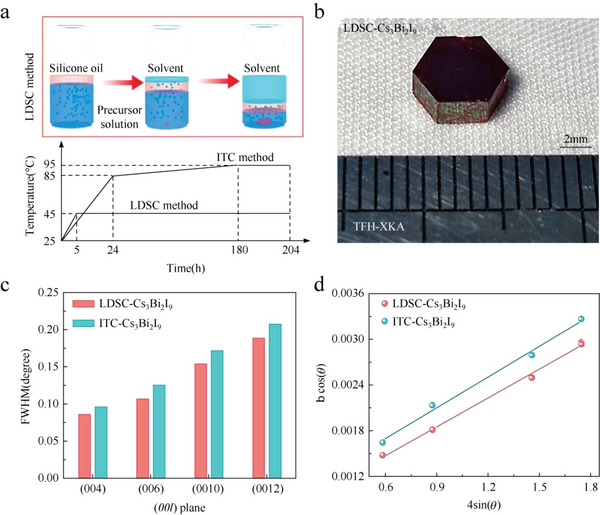
a) Schematic illustration of LDSC. b) The photograph of the as‐prepared Cs_3_Bi_2_I_9_ single crystal. c) The FWHM of the (00*l*) peaks and d) Williamson–Hall plots of the Cs_3_Bi_2_I_9_ single crystal. Reproduced with permission.^[^ ^72^ ^]^ Copyright 2021, Wiley‐VCH GmbH.

In the LDSC method, the antisolvent with lower density is added to the precursor solution, resulting in the gradual decrease of the solubility, and then its crystallization. Compared with the temperature gradient method, the single crystal prepared by this method has lower crystal defect states because it is grown at a constant low temperature. The liquid phase diffusion method for the growth of single‐crystal halide perovskite was first proposed by Yao and his colleagues.^[^ ^71^ ^]^ Later, Wei et al. optimized the growth strategy.^[^ ^72^ ^]^ They added CsI and BiI_3_ with a molar ratio of 3:2 to the DMF solvent. The precursor is fully dissolved by magnetic stirring at room temperature and then filtered. Then, silicone oil with a density between the precursor and DMF solvent is added to the precursor solution (**Figure** **5**a). DMF will spontaneously diffuse through silicone oil and the high‐quality Cs_3_Bi_2_I_9_ single crystals can be obtained after maintaining the solution system at the constant temperature of 45 °C for 8 days (Figure 5b). The LDSC method has less temperature fluctuation and convection caused by thermal gradients in the perovskite solution, which can effectively stimulate ordered growth and suppress defects and cracks. Therefore, they synthesized Cs_3_Bi_2_I_9_ single crystal through the inverse temperature crystallization (ITC) method for comparison. The narrower full‐width at half‐maximum (FWHM) and lower microstrain of LDSC‐Cs_3_Bi_2_I_9_ single crystal indicate enhanced crystallinity and little distortion related to defects and impurities (Figure 5c,d).

#### 2.1.5 Solvent Evaporation Technique

**Figure 6:**
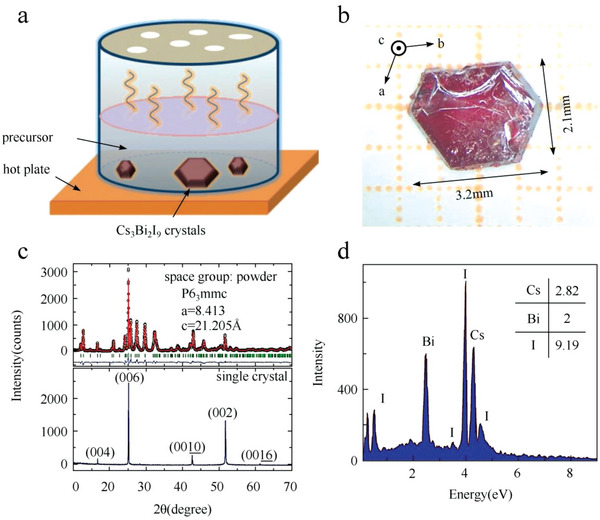
a) The set‐up of preparing perovskite crystal by solvent evaporation. b) The photograph of the as‐prepared Cs_3_Bi_2_I_9_ single crystal. c) XRD patterns of powder and single‐crystal perovskite. d) EDS of Cs_3_Bi_2_I_9_ single crystal. Reproduced with permission.^[^ ^73^ ^]^ Copyright 2018, The Royal Society of Chemistry.

Solvent evaporation is probably the simplest method for single‐crystal perovskite growth. According to this technique, the precursor solution is placed in an open container, and as the solvent slowly evaporates, a supersaturated perovskite solution is formed. The seed crystals spontaneously nucleate and grow into larger crystals at the bottom of the container. Zhang et al. developed an optimized growth strategy to obtain several millimeter‐sized single crystals of Cs_3_Bi_2_I_9_.^[^ ^73^ ^]^ As shown in **Figure** **6**a, the precursor solution was placed in a container, and the top of the container was sealed by aluminum foil with holes. The container was then placed on a hot plate at 60 °C, at which temperature the solubility of Cs_3_Bi_2_I_9_ increased. By volatilizing the solvent, the precursor became saturated again and then cooled to 50 °C at a rate of 1 °C h^−1^. During the cooling process, nuclei were formed and finally grown at 50 °C for 3 days. Figure 6b shows the as‐prepared Cs_3_Bi_2_I_9_ crystal with a hexagonal shape. The rough surface of the crystal is mainly due to the dislocation growth mechanism and the rapid growth rate. The powder X‐ray diffraction (XRD) pattern demonstrates that Cs_3_Bi_2_Br_9_ perovskite belongs to the space group *P6_3_/mmc* and single crystal exhibits only a set of (00l) diffraction faces. The corresponding energy‐dispersive X‐ray spectroscopy (EDS) analysis demonstrates that the ratio of Cs, Bi, and I is close to 3:2:9 (Figure 6c,d). Due to the difference of carrier mobility, the crystal exhibits anisotropic resistivities along the [*00l*] and [*l00*] directions, with values of 1.108 × 10^10^ and 7.7 × 10^8^ Ω cm, respectively, which is beneficial to develop anisotropic X‐ray detectors. More recently, Xu et al. dissolved (Gua)_2_CO_3_ and Bi_2_O_3_ in HI aqueous solution and methanol (CH_3_OH) antisolvent.^[^ ^74^ ^]^ Upon the slow evaporation of the HI/CH_3_OH solvent, the bulk 0D guanidinium bismuth iodide ((Gua)_3_Bi_2_I_9_) single crystals with the sizes of up to 5 × 5 × 0.6 mm^3^ were obtained at room temperature. The low defect state density of 3.35 × 10^10^ cm^−3^ and a large bulk resistivity of 3.94 × 10^11^ Ω cm ensure low noise characteristics in the operating conditions of the X‐ray detector, thus effectively reducing the detection limit.

Antisolvent crystallization and solvent evaporation are the simplest methods for preparing single‐crystal perovskites, which are almost not limited by the experimental equipment. The Bridgman technique is used to prepare large size single‐crystal ingots, which has significant advantages for fabricating wafer‐level X‐ray detector arrays. However, the main drawback of the antisolvent crystallization, solvent evaporation technique, and Bridgman technique is the slow growth rate, typically taking several days. For this reason, most of the works on single‐crystal‐based X‐ray detectors followed alternative growing technique. Temperature gradient method permits rapid solution growth of high‐quality size‐ and shape‐controlled perovskite single crystals. It produces perovskite single crystals with 10 mm in several hours. Therefore, the vast majority of single‐crystal perovskites reported for direct X‐ray detection have been grown through temperature gradient crystallization. In the process of growing single‐crystal perovskite by temperature gradient method, the convective currents caused by thermal gradients inevitably disturb the ordered growth, leading to unfavorable twining defects and cracks in the single‐crystal perovskite. The liquid diffused separation for preparing crystals at room temperature effectively avoids problems such as crystal phase transitions and thermal convection caused by temperature gradients, and the quality of as‐prepared crystals is usually relatively high.

### 2.2 Polycrystalline Film

#### 2.2.1 Spin‐Coating Method

The spin‐coating process is a method of uniformly dispersing the precursor solution on the surface of the substrate rotating at high speed. Perovskite grains are generated from the precursor solution after solvent evaporation, supersaturation, and post‐annealing. Therefore, the film quality is fundamentally affected by the properties of the substrate material, the ratio of the perovskite precursor solution, the parameters of the spin coating process, and the annealing temperature conditions.^[^ ^75^, ^76^ ^]^ Although the one‐step solution method has the potential for commercial large‐scale preparation due to the simple operation and equipment requirements, the obtained thin films are very detrimental to the optoelectronic properties of perovskites due to their roughness and low coverage to the substrate. Generally, the antisolvent engineering and multistep spin‐coating process can optimize film quality.^[^ ^77^, ^78^ ^]^

**Figure 7:**
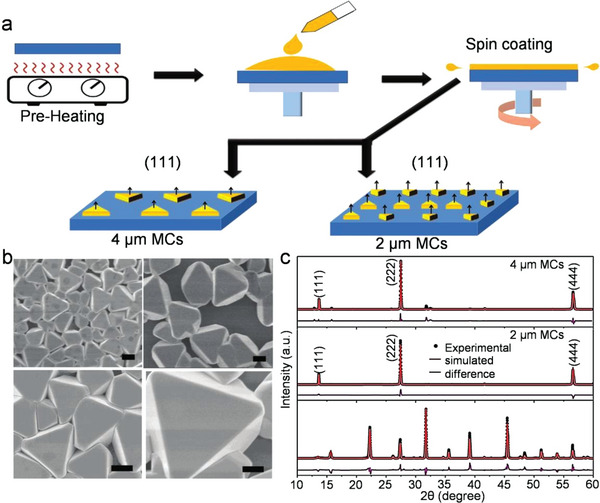
a) Spin‐coating method for fabricating Cs_2_AgBiBr_6_ MCs. b) As‐prepared MCs with the 2 µm (left) and 4 µm (right) size. c) XRD patterns of Cs_2_AgBiBr_6_ thin film, 2 and 4 µm MCs. Reproduced with permission.^[^ ^79^ ^]^ Copyright 2021, Wiley‐VCH GmbH.

Hossain et al. developed a simple hot‐spin casting method for synthesizing the highly crystalline Cs_2_AgBiBr_6_ microcrystalline films with highly packed (111) planes.^[^ ^79^ ^]^ During spin coating, preheating induces the process of slow solvent evaporation to form the supersaturated perovskite solution, which stimulates the formation of heterogenous nucleation centers (**Figure** **7**a). The perovskite microcrystals (MCs) of 2 µm size are formed without post‐annealing and the size increases to 4 µm by employing a two‐step spin‐coating process (Figure 7b). Interestingly, there is a strong facet orientation of the as‐prepared perovskite sample. The XRD patterns of both 2 and 4 µm MCs contain only (111) planes and belong to the cubic phase with space group *Fm‐3m* (Figure 7c). Afterward, Achoi et al. proposed a method to prepare pinhole‐free methylammonium bismuth iodide (MA_3_B_2_I_9_) thin films by multistep spin coating.^[^ ^80^ ^]^ By optimizing the number of spin‐coating layers, the prepared perovskite layer has no pinholes, which can reduce the adverse effects of pinholes generated during the spin‐coating process.

#### 2.2.2 Inkjet Printing, Spray‐, Blade‐, and Slot‐Die Coating

Similar to digital printers, in inkjet printing, droplet size and trajectories are finely controlled through nozzles, usually piezoelectric microelectromechanical systems (MEMS) printheads, located in close proximity to the substrate to achieve ultrafine lateral resolution. The commercialization advantage of the inkjet printing process is that the perovskite can be directly patterned, so the material utilization rate is high.^[^ ^81^, ^82^ ^]^

Spraying is a process in which a subjected fluid is mechanically driven to produce atomized droplets that fall onto the surface of a substrate, either naturally or by being guided by a carrier gas.^[^ ^83^, ^84^ ^]^ Due to the randomness of droplet size and location, and the potential for new droplets to dissolve already deposited material, it is important to maintain coverage during spraying while maintaining a high substrate temperature to inhibit the redissolution process.

**Figure 8:**
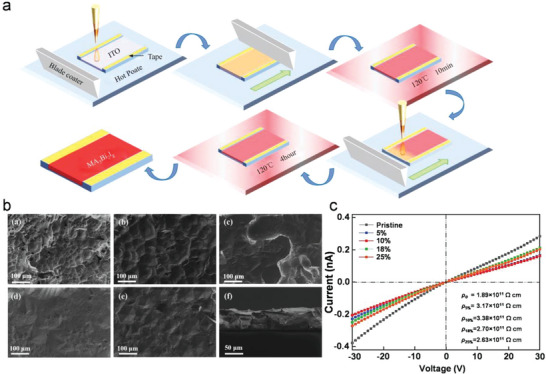
a) Schematic diagram of the preparation of MA_3_Bi_2_I_9_ films by blade coating. b) SEM images of MA_3_Bi_2_I_9_ films with 0%, 5%, 10%, 18%, and 25% MACl additive. c) The resistivity of MA_3_Bi_2_I_9_ films. Reproduced with permission.^[^ ^87^ ^]^ Copyright 2022, The Royal Society of Chemistry.

Blade coating is a technique in which a knife‐type coating tool is moved over the surface of the substrate, dispersing the solution on the substrate and removing excess solution (**Figure** **8**a). Blade coating is a scalable, simple, and low‐cost method. Compared with spin coating, the solvent volatilization process is much slower, which would result in higher coverage and better quality of perovskites.^[^ ^85^, ^86^ ^]^ In addition, the blade shape can customize the blade angle and shape to meet the needs of various scenarios. In 2022, Dong et al. explored green methylammonium acetate solvent blade‐coated MA_3_Bi_2_I_9_ films, methylamine chloride (MACl) with different proportions (Cl/I = 5%, 10%, 18%, 25%) was used to study the influence on the crystal quality of the films.^[^ ^87^ ^]^ They find that the existence of MACl delays the crystallization rate and significantly changes the micromorphology of the film (Figure 8b). The fabricated MA_3_Bi_2_I_9_ films show the highest resistivity of 3.38 × 10^11^ Ω cm with the 10% MACl additive (Figure 8c).

**Figure 9:**
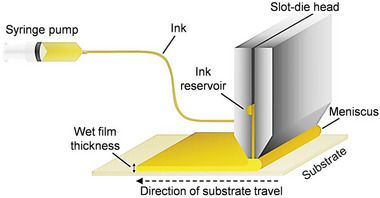
Schematic of a slot‐die coating process. Reproduced with permission.^[^ ^88^ ^]^ Copyright 2019, Elsevier Ltd.

The process of slot‐die coating is similar to blade‐coating, and the coating solution is dispersed to the substrate by filling the gap between the die and the substrate using an ink container with slits (**Figure** **9**).^[^ ^88^ ^]^ The quality of a slot‐die‐coated film can be affected by the viscosity of the liquid, coating speed, knife, and substrate clearance. The commercial advantages of the slot‐die coating method are the potential for large‐area production with little solution waste and compatibility with continuous roll‐to‐roll technology.

#### 2.2.3 Physical/Chemical Vapor Deposition Method

Physical vapor deposition (PVD) and chemical vapor deposition (CVD) are described as chemical reaction processes carried out by specific chemical species directed to a substrate by an inert transport gas under given pressure, temperature, and plasma conditions.^[^ ^89^, ^90^, ^91^ ^]^ Usually, a tubular flow reactor surrounded by a heating furnace can conveniently control the temperature to complete the continuous deposition process. The advantage of this method is that the thickness control is more accurate, and it allows the introduction of perovskite components (e.g., MAI and BiI_3_) from the powder or gas phase.

**Figure 10:**
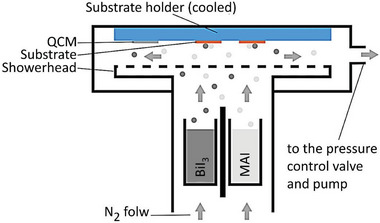
Schematic illustration of the CVD tool for synthesizing lead‐free perovskite films. Reproduced with permission.^[^ ^95^ ^]^ Copyright 2019, Springer Nature.

At present, thin films of various perovskite materials have been successfully deposited by one‐step CVD under controlled pressure, paving the way for large‐area production.^[^ ^92^, ^93^, ^94^ ^]^ For example, Sanders et al. reported a novel CVD process for air‐stable Pb‐free methylammonium bismuth iodide films, which enabled a homogenous deposition on large‐area substrates up to 108 cm^2^ using close‐coupled showerhead technology.^[^ ^95^ ^]^ A schematic of the CVD equipment is shown in **Figure** **10**. The evaporation source consists of BiI_3_ and MAI, which are separated by a shield to avoid cross‐contamination. A quartz crystal microbalance was used to measure the deposition rate. Most of the reported high‐quality perovskite films deposited by CVD use multistep CVD, as well as CVD combined with spin coating and thermal evaporation, which are of higher quality than those deposited by one‐step methods. However, it is also more difficult to control the stoichiometry of the deposited films, increasing the cost of the manufacturing process.

The preparation techniques of polycrystalline can be divided into solution‐based and vapor‐based methods. Spin‐coating, inkjet printing, spray‐, blade‐, and slot‐die coating are the main solution‐based scalable deposition methods and vapor‐based methods mainly include PVD and CVD. The solution‐based deposition techniques are widely used for the realization of perovskite polycrystalline thick/thin films. Spin‐coating is a facile solution‐based deposition technique, which can be combined with photolithography to obtain the patterned perovskite film. The printing, spray‐, blade‐, and slot‐die coating techniques are intended as the additive solution‐based deposition processes enabling large‐area thick‐film preparation, showing significant advantages in the preparation of wafer‐scale perovskite X‐ray detectors. Although the solution‐based method has great advantages, pinholes will produce during solvent evaporation and deteriorate the performance of X‐ray detectors. Vapor‐based deposition is a solvent‐free method that it is easy to prepare high‐quality, uniform films on a variety of substrates. Because the performance of X‐ray detector is greatly affected by perovskite material, the preparation technology of materials can be optimized to achieve the preparation of high‐performance detectors.

## 3 Fundamental Principles

### 3.1 Detection Mechanism

**Figure 11:**
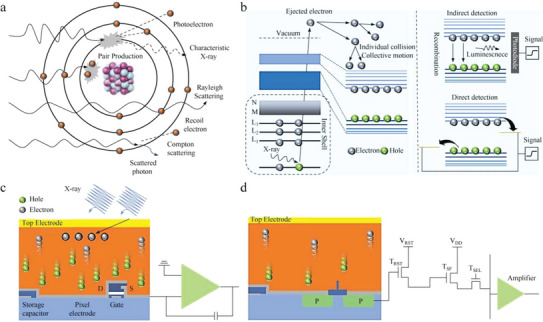
a) The interactions between photons and substances. b) The mechanism of electron–hole generation and detection mechanism of radiation detectors. c) Device configuration of readout integrated circuit based on TFT and d) device configuration of CMOS readout array for direct radiation detectors. Reproduced with permission.^[^ ^97^ ^]^ Copyright 2020, Elsevier Inc.

There are four main types of interaction between photons and semiconductor materials: photoelectric effect, Rayleigh scattering, Compton scattering, and pair production.^[^ ^96^ ^]^ In the photoelectric effect, photons are completely absorbed by atoms and photocarriers are emitted. It is an important mechanism of radiation detectors. During Compton scattering, photons collide with free electrons and transfer part of their energy to electrons (**Figure** **11**a). High energy electrons in the inner shell, which are produced by the interaction between photons and substances, move through the semiconductor and release many low‐energy free electrons. The free electrons then lift into the conduction band, while the holes migrate to the top of the valence band. The generated electron–hole pairs can be collected by electric field or emit visible photons in a radiation recombination (Figure 11b).^[^ ^97^ ^]^ For direct radiation detector, photons can be converted into many excess free charges and then directly collected by electrodes under external bias.

The device structure and a single‐pixel structure of the thin‐film transistor (TFT) readout circuit are shown in Figure 11c. Radiation photons are absorbed by the semiconductor and carriers are generated. The pixel electrode collects charges and stores them in the storage capacitor. When TFT turns on, charges are transferred from the capacitor to the charge amplifier and transformed into a voltage signal, which is quickly processed by a readout integrated circuit. A single‐pixel structure of the complementary metal‐oxide semiconductor (CMOS) readout circuit is shown in Figure 11d, which includes a detector, a charge transfer gate (*T*_x_), a reset transistor, a source following transistor, and a row selection transistor. Under irradiation, photogenerated electrons are collected in a surface potential well. Before integration, the floating diffusion output is reset by the reset transistor first, and the reset voltage is sampled by the source follower output. When the signal integration stage, the control signal is from high level to low level and the signal charge is transferred to the floating diffusion output. A voltage signal by subsequent circuit sampling again, twice the difference between the sampling signal is the final signal.

### 3.2 Performance Parameters

#### 3.2.1 Dark Current

The dark current, which is related to dark shot noise, mainly comes from the existence of defective states, thermal charge carrier generation, and the injection of carriers at the interface of the electrodes. Accordingly, the dark current density (*J*_d_) is defined as the dark current flowing through the device per unit area. For practical applications, minimizing the *J*_d_ is crucial to improve important parameters such as the signal‐to‐noise ratio, sensitivity, and limit of detection.

The presence of ion migration causes the dark current and photocurrent baseline drift, and the current drift *D* can be calculated using the following equation

(1) $$D(A\;cm^{-1}\;s^{-1}\;V^{-1})\;=\;(J_{d1}-J_{d2})/tE$$

where *J*_d1_ and *J*_d2_ are the starting and ending current densities, respectively. *t* is the duration time and *E* is the operating electric field.

#### 3.2.2 Sensitivity

Detection sensitivity (*S*) is another important parameter of an X‐ray detector, which represents the response ability of the detector to a specific dose rate. Increasing the carrier mobility‐lifetime (*µτ*) product and working electric field can effectively improve the sensitivity. Considering that a large working electric field will enhance the charge trapping and de‐trapping processes, leading to a poor detection limit. Therefore, optimizing the crystal quality and increasing *µτ* product are the most fundamental way to improve the sensitivity. The *S* can be defined by the following equation

(2) $$S\left(\mu C\;{\mathrm{Gy}}_{\mathrm{air}}^{-1}\;{\mathrm{cm}}^{-2}\right)=\left(I_{p}-I_{d}\right)/D\cdot A$$

where *I*_p_ and *I*_d_ are the photocurrent and dark current, respectively. *D* is the dose rate and *A* is the effective area.

#### 3.2.3 Detection Limit

In the medical field, most medical diagnostic systems require a dose rate of less than 5.5 µGy s^−1^. In order to reduce the harm of radiation dose to the human body, the detection limit is an important figure of merit. The detection limit defined the dose rate with a signal‐to‐noise ratio (SNR) value of 3 at a given voltage. Then the SNR is calculated as: SNR = *I*_signal_/*I*_noise_. The signal current (*I*_signal_) is obtained by subtracting the average dark current from the average photocurrent (*I*_signal_ =$\bar{I}\;$_photo_−$\bar{I}$_dark_) and the noise current (*I*_noise_) is obtained by calculating the standard deviation of the photocurrent

(3) $$I_{\mathrm{noise}}=\sqrt{\frac{1}{N}\sum_{i}^{N}{\left(I_{i}-{\bar{I}}_{\mathrm{photo}}\right)}^{2}}\;$$

It is evident that the detection limit strongly depends both on the X‐ray response signal amplitude and the noise of the detector.

#### 3.2.4 Response Time

In general, response time quantifies the speed of detectors, which includes the rise time and fall time. The latter is often longer because it is more sensitive to the long de‐trapping process of defects in the perovskite material. Short response time is desirable for X‐ray detectors to minimize the time of X‐ray exposure of the patients. The rise time and fall time are defined as the time experienced by the detector from 10% to 90% and from 90% to 10% of the maximum signal value, respectively.

#### 3.2.5 Spatial Resolution

For X‐ray imaging, spatial resolution is an extremely important factor, which refers to the ability to distinguish two adjacent features. The spatial resolution is affected by the detector pixel size, material, radiation dose, and other factors. Detectors with high spatial resolution can reliably detect micron‐scale cancerous lesions or fractures. The spatial resolution is measured in line pairs per centimeter (lp cm^−1^) based on the image, i.e., the maximum number of stripes and gaps per centimeter can be distinguished visually. A line pair consists of a line and a space, and the width of the space is equal to the width of the line. The more the pairs of lines that can be distinguished clearly within the range of unit width, the higher the spatial resolution. The conversion relationship is: the minimum recognizable object diameter (mm) = 5÷lp cm^−1^. The spatial resolution can be directly measured with the resolution test card. While this intuitive approach is widely accepted, there is a more scientific approach. The modulation transfer function (MTF) can be used to describe spatial resolution. By plotting MTF versus line pairs per centimeter, spatial resolution is defined as the line pairs per centimeter at a specific contrast.

#### 3.2.6 Gain Factor

The gain factor (*G*) represents the charge collection capacity of the X‐ray detector, which can be calculated as follows: *G* = *I*_R_/*I*_P_, where *I*_R_ is the measured current, and *I*_P_ is the theoretical current, which is defined as *I*_p_ = *φβe*, where *φ* is the photon absorption rate (photons s^−1^), and *β* is the maximum number of photogenerated carriers per photon. *φ* = *ɛDm*_s_/*E*_ph_, where *D* is the dose rate, *m*_s_ is the sample mass, *ɛ* is the fraction of absorbed photons, and *E*_ph_ is the X‐ray energy. *β* = *E*_ph_/Δ, where Δ is the empirical ionization energy.

Therefore, the theoretical current *I*_P_ can be calculated by the equation: *I*_p_ = *φβe* = *ɛDm*_s_ *e*/Δ.

## 4 Typical Lead‐Free Perovskite X‐Ray Detectors

Excellent perovskite X‐ray detectors usually contain the following characteristics: high absorption coefficient, large *µτ* product, tunable optical band gap, and easy‐processing. In addition, the high defect tolerance of perovskite mitigates the effect of defects of significant density, thus preserving the properties of functional materials, which has the positive effects on the X‐ray detector performance, such as minimization of ghosting artifacts. Bismuth (Bi) is the heaviest element, which can produce effective X‐ray absorption. Double halide perovskites with the Bi^3+^‐Ag^+^ system, especially the Cs_2_AgBiBr_6_, and the low‐dimensional Bi‐based family are the two most representative types for direct X‐ray detection. Besides, we have also discussed lead‐free Te/Sb‐based perovskite X‐ray detectors.

### 4.1 Bi^3+^‐Ag^+^‐Based Perovskite X‐Ray Detectors

Among the double halide perovskite family, Bi^3+^‐Ag^+^‐based double perovskite is one of the most intriguing and has emerged as a promising and potential alternative to lead‐based perovskites for radiation detection due to indirect bandgap, long carrier lifetime, and excellent stability. And Cs_2_AgBiBr_6_ is the most representative among the double perovskites and a research hotspot.^[^ ^98^, ^99^ ^]^ We have summarized the major parameters of Bi^3+^‐Ag^+^‐based X‐ray detectors as listed in **Table** **1**. The reported sensitivity is generally lower than that of lead‐containing perovskites, but more stable than organic–inorganic hybrid perovskites. More excitedly, the (BA)_2_CsAgBiBr_7_/Cs_2_AgBiBr_6_ heterojunction allows the formation of built‐in electric field, enabling the device to achieve a detection sensitivity of 206 µC Gy_air_^−1^ cm^−2^ at 0 V and low dark current drift 6 × 10^−5^ nA cm^−1^ s^−1^ V^−1^.

**Table 1:** Device performance of Bi^3+^‐Ag^+^‐based perovskite X‐ray detectors

Device structure	Electric field [V mm^−1^]	Sensitivity [µC Gy_air_^−1^ cm^−2^]	Detection limit [nGy_air_ s^−1^]	Ref.
Au/Cs_2_AgBiBr_6_ SC/Au	2.5	105	59.7	[99]
Au/Cs_2_AgBiBr_6_ wafer/Au	500	250	95.3	[100]
Au/Cs_2_AgBiBr_6_ SC/Au	50	1974	45.7	[56]
W/Cs_2_AgBiBr_6_ film/Pt	109	487	–	[101]
Au/(BA)_2_CsAgBiBr_7_/Au	5	4.2	–	[102]
Au/(BA)_2_CsAgBiBr_7_/Cs_2_AgBiBr_6_/Au	0	206	–	[103]
Au/ (DFPIP)_4_AgBiI_8_/Au	1.25 × 10^3^	188	1.58 × 10^3^	[104]
Ag/(I‐C_4_H_8_NH_3_)_4_AgBiI_8_/Ag	≈4.55	5.38	–	[105]
Au/Cs_2_AgBiCl_6_/Au	40	325.78	241	[106]

Due to the requirement of the large thickness and low defect state density in high‐performance radiation detectors, the quality and the size of the Cs_2_AgBiBr_6_ single crystal are important. Yin et al. employed solubility and supersolubility as quantitative indicators to direct the growth of Cs_2_AgBiBr_6_ single crystals.^[^ ^56^ ^]^ The increase of *µτ* product verifies the improved crystallinity and reduced defect state within the crystals by following a controlled cooling process. The sample from controlled cooling preparation has a lower dark current of about 310 pA and a slightly higher photocurrent of around 120 pA. The detector shows a sensitivity of 24.23 µC Gy_air_^−1^ cm^−2^ under 2.5 V mm^−1^ electric field and the value further reaches 1974 µC Gy_air_^−1^ cm^−2^ under 50 V mm^−1^ electric field.

**Figure 12:**
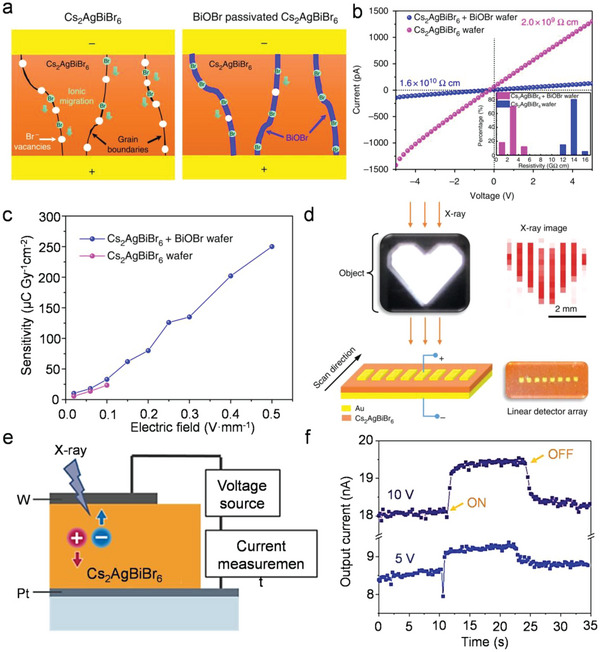
a) Schematic illustration of ion migration inhibition by BiOBr passivation. b) The resistivity of the devices with/without passivation. c) Device sensitivity‐dependent applied electric fields. d) X‐ray image of the heart‐shaped logo. Reproduced with permission.^[^ ^100^ ^]^ Copyright 2019, Springer Nature. e) Schematic of X‐ray detection measurements. f) X‐ray time response of Cs_2_AgBiBr_6_ device. Reproduced with permission.^[^ ^101^ ^]^ Copyright 2021, American Chemical Society.

Perovskite film with the thickness more than hundreds of micrometers is a prerequisite for complete X‐ray attenuation. Currently, growing single‐crystal perovskites with a certain thickness and shape is still a great challenge. The tablet wafer is not only size‐controllable but also time saving in large‐scale production than single crystals, which has been widely studied. Due to the polycrystalline nature, there are still amounts of grain boundaries within the vast majority of tablet wafer. For the Cs_2_AgBiBr_6_ perovskite, Br^−^ vacancies are the major ionic migration channels, which results in serious baseline drift for the wafer. To inhibit ion migration, Yang et al. passivated the Cs_2_AgBiBr_6_ perovskite wafer by introducing bismuth oxybromide (BiOBr) as heteroepitaxial layers. Br^−^ provided by BiOBr can reduce Br^−^ vacancy in perovskite, thereby inhibiting ion migration and reducing the ionic conductivity (**Figure** **12**a).^[^ ^100^ ^]^ After passivation, the perovskite wafer resistivity increased from 2.0 × 10^9^ to 1.6 × 10^10^ Ω cm (Figure 12b). As shown in Figure 12c, the sensitivity for passivated Cs_2_AgBiBr_6_ wafer‐based X‐ray detector increased from 10 to 250 µC Gy_air_^−1^ cm^−2^, which is higher than that of the pristine Cs_2_AgBiBr_6_ device. The assembled linear array detector with a pixel size of 0.8 mm realizes X‐ray imaging of heart‐shaped logo by linearly scanning in one direction (Figure 12d).

Haruta et al. improved the sensitivity of X‐ray detectors by forming a columnar grain structure in the photoconductive layer in order to decrease grain boundaries.^[^ ^101^ ^]^ They proposed a mist deposition method, which is one of the ultrasonic‐assisted spray deposition methods, for the columnar grain growth of Cs_2_AgBiBr_6_ films. And a high re‐dissolution capability can form a sufficiently wetted region to enhance grain growth. The X‐ray detector with the structure of W/Cs_2_AgBiBr_6_/Pt exhibits a resistivity of 1.0 × 10^10^ Ω cm and an increased X‐ray response, as shown in Figure 12e,f. The sensitivity, which is 208 µC Gy_air_^−1^ cm^−2^ at 5 V and increased to 487 µC Gy_air_^−1^ cm^−2^ at 10 V, is also enhanced by the columnar grain structure compared to the detectors based on Cs_2_AgBiBr_6_ polycrystalline thick films.

**Figure 13:**
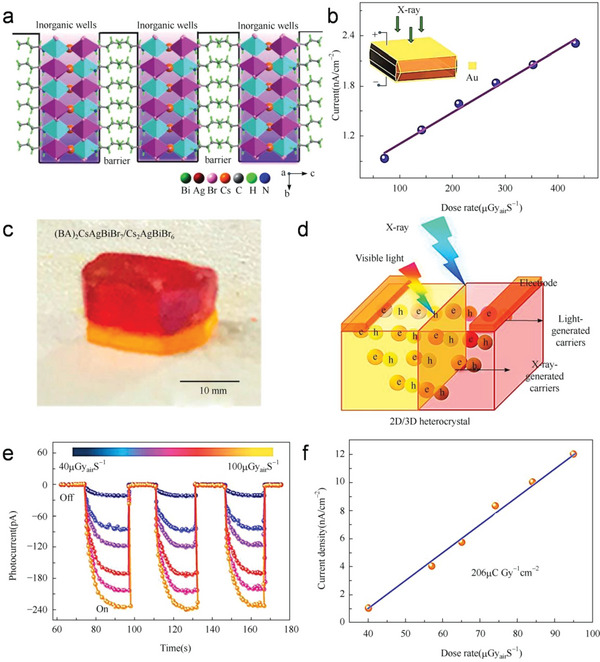
a) Structural configuration of (BA)_2_CsAgBiBr_7_ that defines the 2D perovskite quantum‐confined motif. b) X‐ray‐generated photocurrent at various dose rates under a bias of 10 V. Reproduced with permission.^[^ ^102^ ^]^ Copyright 2019, Wiley‐VCH Verlag GmbH &Co. KGaA. c) As‐prepared (BA)_2_CsAgBiBr_7_/Cs_2_AgBiBr_6_ heterocrystal. d) Diagram of the heterocrystal‐based detector. e) Time‐photocurrent of the heterocrystal‐based detector at *V*_bias_ = 0 V with different dose rates. f) X‐ray photocurrent densities at various X‐ray radiation under zero bias. Reproduced with permission.^[^ ^103^ ^]^ Copyright 2021, American Chemical Society.

In addition to Cs_2_AgBiBr_6_, some other Bi^3+^‐Ag^+^‐based double perovskites are also studied to be used in X‐ray detection. Xu et al. introduced a 2D multilayered (BA)_2_CsAgBiBr_7_ (BA^+^ = n‐butylammonium), as shown in **Figure** **13**a.^[^ ^102^ ^]^ The crystal exhibits a high *µτ* product of 1.21 × 10^−3^ cm^2^ V^−1^, a high bulk resistivity of 1.5 × 10^11^ Ω cm, low density of defects, and traps of 4.2 × 10^10^ cm^−3^. Due to the less effective charge traverse in the out‐of‐plane direction compared with the 3D halide perovskites, the sensitivity of the device is 4.2 µC Gy_air_^−1^ cm^−2^, as shown in Figure 13b. In the solid‐state device, the heterojunction crystal promotes charge transport and suppresses the noise current. Given this, Zhang et al. report a solution‐processed in situ heteroepitaxial approach to integrating the first lead‐free halide perovskite (BA)_2_CsAgBiBr_7_/Cs_2_AgBiBr_6_ heterojunction with near atomically sharp interfaces (Figure 13c).^[^ ^103^ ^]^ During heterojunction growth, trap state, vacancies, and disorders inevitably formed in the crystal, which acts as the carrier recombination center, causing performance degradation. To reduce this effect, the planar‐structure heterocrystal devices are fabricated by coating Au electrodes and the schematic of the device is shown in Figure 13d. X‐ray photoresponse as a function of time under different X‐ray dose rates is shown in Figure 13e. Under zero bias, the device exhibits self‐driving behavior due to its built‐in electric potential. The dark current is as low as 3.2 × 10^−2^ pA, while the photocurrent can reach up to 240 pA, and a sizable on/off switching ratio of 10^4^. By linear fitting of Figure 13f, an impressive sensitivity of 206 µC Gy^−1^ cm^−2^ is derived from the slope.

Wang et al. reported a 2D (DFPIP)_4_AgBiI_8_ (DFPIP = 4,4‐difluoropiperidinium) for X‐ray ferroelectric devices.^[^ ^104^ ^]^ The crystal structure is similar to A_2_PbX_4_, and the bilayers of DFPIP cations are embedded between the inorganic layers. Benefitting from the ring‐like DFPIP cation, (DFPIP)_4_AgBiI_8_ exhibits an excellent ferroelectricity as *T*_c_ = 422 K and *P*_s_ = 10.5 µC cm^−2^, while the 2D (AgBiI_8_)^4−^ anion layer enhances the X‐ ray photoresponse and makes (DFPIP)_4_AgBiI_8_ device achieve a sensitivity up to 188 µC Gy_air_^−1^ cm^−2^ and a detection limit of 3.13 µGy_air_ s^−1^, which is much better than previously reported X‐ray‐sensitive ferroelectric material. Xu et al. also reported (I‐ BA)_4_AgBiI_8_ (I‐BA^+^ = I‐n‐butylammonium) single crystals for X‐ray detection with a similar structure to (DFPIP)_4_AgBiI_8_.^[^ ^105^ ^]^ The crystal exhibits a low trap density of 3.78 × 10^10^ cm^−3^ and a high *µτ* product of 2.28 × 10^−3^ cm^−2^ V^−1^. Benefitting from these, X‐ray detectors based on (I‐BA)_4_AgBiI_8_ single crystals possess a sensitivity of 5.38 µC Gy_air_^−1^ cm^−2^ and photocurrent increased linearly as the dose rate increased from 0.5 to 2.7 mGy s^−1^. Most recently, the fabricated vertical structured Cs_2_AgBiCl_6_ X‐ray detector exhibits self‐powered behavior and long‐term ambient storage.^[^ ^106^ ^]^

### 4.2 Low‐Dimensional Bi‐Based Perovskite X‐Ray Detectors

A_3_Bi_2_X_9_ perovskite can be divided into organic–inorganic hybrid and all‐inorganic perovskite according to the type of A‐site cation. For the organic–inorganic hybrid perovskite, recent research focuses on the hybrid 0D (MA)_3_Bi_2_I_9_ (MA^+^ = CH_3_NH_2_^+^). Besides, all‐inorganic perovskites such as Cs_3_Bi_2_I_9_, Rb_3_Bi_2_I_9_, and (NH_4_)_3_Bi_2_I_9_ perovskites are also used to construct X‐ray detectors. **Table** **2** summarizes the basic parameters of low‐dimensional Bi‐based perovskite X‐ray detectors. It is clear that the MA_3_Bi_2_I_9_ perovskite X‐ray detectors exhibit high sensitivity and low detection limit. Furthermore, there is no deterioration in detection performance when these X‐ray detectors are exposed to high radiation doses, which guarantee reliable operation for radiation detection application.

**Table 2:** Device performance of low‐dimensional Bi‐based perovskite X‐ray detectors

Device structure	Applied electric field [V mm^−1^]	Sensitivity [µC Gy_air_^−1^ cm^−2^]	Detection limit [nGy_air_ s^−1^]	Stability	Ref.
Au/Cs_3_Bi_2_I_9_/Au	20	4382	7.93	–	[33]
Au/Cs_3_Bi_2_I_9_/Au	50	1652.3	130	2.8 Gy_air_	[55]
Au/(NH_4_)_3_Bi_2_I_9_ Au/	2.2	8.2 × 10^3^	210	–	[57]
Au/MA_3_Bi_2_I_9_/Au	48	10 620	0.62	23.8 Gy_air_	[63]
Au/(Gua)_3_Bi_2_I_9_/Au	≈833.3	18.23	237.54	–	[74]
Au/MA_3_Bi_2_I_9_/Au	210	563	9.3	172 Gy_air_	[107]
Au/MA_3_Bi_2_I_9_/Au	–	2065	2.71	107.59 Gy_air_	[108]
Au/Cs_3_Bi_2_I_9_/Au	45	111.9	–	–	[109]
Cs_3_Bi_2_I_9_/MXene	120	368	231	237.8 mGy_air_	[110]
Au/Rb_3_Bi_2_I_9_/Au	300	159.7	8.32	480 000 Gy_air_	[111]
Au/AgBi_2_I_7_/Au	0.38	282.5	72	58 Gy_air_	[112]

Zheng et al. employed the seed‐crystal‐assisted constant‐temperature evaporation method to grow 0D MA_3_Bi_2_I_9_ single crystal with a size of 27 mm × 23 mm × 13 mm.^[^ ^63^ ^]^ The bulk single crystal exhibits anisotropy in electrical properties due to the different orientation arrangement of (Bi_2_I_9_)^3−^ clusters, and the results demonstrate the crystals cut along the *c*‐axis show a resistivity of 5.27 × 10^11^ Ω cm, which is much higher than that of the crystals cut perpendicular to the *c*‐axis, and a lower charge carrier *µτ* product of 1.2 × 10^−3^ cm^2^ V^−1^. The detector fabricated with the structure of Au/MA_3_Bi_2_I_9_/Au exhibits excellent operational stability, a dark current density of 0.98 nA cm^−2^, a detection limit (LoD) of 0.62 nGy_air_ s^−1^, and an X‐ray sensitivity of 10 620 µC Gy_air_^−1^ cm^−2^ at the operating bias of 120 V benefitting from the better electrical properties of the crystals cut along the *c*‐axis, such as the higher resistivity, the high activation energy of 0.46 eV for ion migration, and a low dark carrier concentration of 10^6^ cm^−3^. Instead of bulk crystals, Tie et al. reported MA_3_Bi_2_I_9_ polycrystalline pellets (PPs) with good ambient and thermal stability that can be facilely prepared by directly grinding the MA_3_Bi_2_I_9_ crystal and then compressed into a polycrystalline wafer by cold isostatic‐pressing.^[^ ^107^ ^]^ MA_3_Bi_2_I_9_‐PPs exhibit a strong X‐ray absorption capability and the photon energy‐dependent absorption coefficient. The Au/MA_3_Bi_2_I_9_‐PPs/Au device possesses a stable signal response to continuous X‐ray pulse owing to the low and stable dark current. Besides, the device also exhibits a low LoD of 9.3 nGy_air_ s^−1^, and the sensitivity of 563 µC Gy_air_^−1^ cm^−2^ is much higher than that of the *a*‐Se detector.

**Figure 14:**
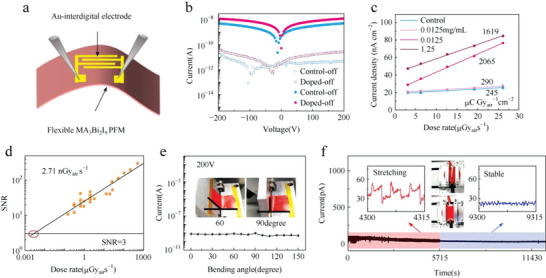
a) Device structure of coplanar X‐ray MA_3_Bi_2_I_9_ detectors. b) *I*–*V* curves of the control and doped (0.125 mg mL^−1^) detectors with X‐ray on and off. c) Current densities at different dose rates. d) SNR of the detector at 200 V bias. e) The current of the detector at a different bending angle. f) *I*–*T* response of the detector during the stretching test. Reproduced with permission.^[^ ^108^ ^]^ Copyright 2022, Wiley‐VCH GmbH.

Flexible electronic devices have potential application advantages in the wearable field, which have aroused extensive interest in the research field.^[^ ^113^, ^114^, ^115^ ^]^ Liu et al. have designed a flexible X‐ray detector based on the perovskite MA_3_Bi_2_I_9_ polycrystalline film, which is comparable to the single‐crystal X‐ray detector in sensitivity and detection limit.^[^ ^108^ ^]^ Devices with a coplanar Au‐interdigital structure are shown in **Figure** **14**a. Without F4‐TCNQ (2,3,5,6‐tetrafluoro‐7,7,8,8‐tetracyanoquinodimethane) doping, the control device exhibits the minimum dark current away from 0 V due to high ion migration in perovskite (Figure 14b). The F4‐TCNQ treatment (0.0125 mg mL^−1^) promotes the charge extraction at the MA_3_Bi_2_I_9_/Au interface and the sensitivity can reach 2065 µC Gy_air_^−1^ cm^−2^ (Figure 14c), which is the highest among all environmentally friendly flexible X‐ray detectors. As shown in Figure 14d, the low detection limit (2.71 nGy_air_ s^−1^) is more than 2000 times lower than that of regular medical diagnostics (5.5 µGy_air_ s^−1^). The current variation of the F4‐TCNQ‐doped device is independent of bending time and bending angle (Figure 14e), as well as acting over 1000 stretching cycles (0–5715 s) without any significant current decay (Figure 14f). Under the total dose of 107.59 Gy_air_, the device performance has no deterioration, which indicates the excellent irradiation stability.

Besides, Xu et al. reported 0D guanidinium bismuth iodide ((Gua)_3_Bi_2_I_9_) single crystals with a similar crystal structure to MA_3_Bi_2_I_9_ via the antisolvent‐assisted evaporation crystallization method.^[^ ^74^ ^]^ The crystal has a high bulk resistivity of 3.94 × 10^11^ Ω cm. The absorption coefficient of (Gua)_3_Bi_2_I_9_ is higher than *α*‐Se and comparable to CsPbBr_3_, while the attenuation efficiency is larger than *α*‐Se and MAPbBr_3_, and comparable to CsPbBr_3_. And the vertical Au/(Gua)_3_Bi_2_I_9_/Au X‐ray detector exhibits a sensitivity of 18.23 µC Gy_air_^−1^ cm^−2^ at a bias voltage of 500 V, an LoD of 237.54 nGy_air_ s^−1^, and better thermodynamic stability due to the substitution of Gua^+^ for MA^+^.

**Figure 15:**
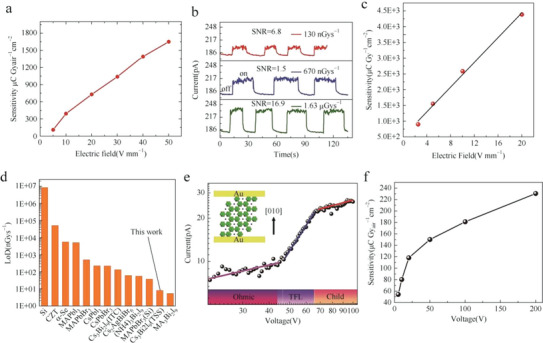
a) Sensitivity under different electric fields of the Cs_3_Bi_2_I_9_ PSC X‐ray detector. b) X‐ray photocurrent response of the Cs_3_Bi_2_I_9_ PSC device under electric fields of 50 V mm^−1^ when exposed to different X‐ray dose rates. Reproduced with permission.^[^ ^55^ ^]^ Copyright 2020, Springer Nature. c) Sensitivity changes of X‐ray detector under different bias voltages. d) The detection limits of X‐ray detectors based on different materials are compared with this work. Reproduced with permission.^[^ ^33^ ^]^ Copyright 2022, American Chemical Society. e) Dark current–voltage characteristic of the (−120) plane is measured using the SCLC method along the [010] direction. Inset: a sandwich structure of Au/Cs_3_Bi_2_Br_9_ SC/Au. f) X‐ray sensitivity of the optimized Cs_3_Bi_2_Br_9_ SC for different voltages. Reproduced with permission.^[^ ^70^ ^]^ Copyright 2021, Science China Press and Springer‐Verlag GmbH Germany, part of Springer Nature.

For all‐inorganic A_3_Bi_2_X_9_ perovskite, Cs_3_Bi_2_I_9_ has aroused much attention for its good properties and excellent stability, and the enhancement of Cs_3_Bi_2_I_9_ single‐crystal quality can lead to the increase of properties of the X‐ray detectors.^[^ ^116^ ^]^ To get high‐quality crystals, Zhang et al. reported a nucleation‐controlled solution method to prepare large size and high‐quality Cs_3_Bi_2_I_9_ perovskite single crystals with a similar crystal structure to MA_3_Bi_2_I_9_.^[^ ^55^ ^]^ The Tauc plot calculated displays a band gap of 1.96 eV, and the trap density is calculated to be 1.4 × 10^10^ cm^−3^. Benefitting from the low trap density, wide bandgap, and high resistivity of 2.79 × 10^10^ Ω cm, a low dark current can be obtained and leads to a low detection limit. The absorption coefficient of Cs_3_Bi_2_I_9_ is higher than the traditional inorganic semiconductor materials, and Cs_3_Bi_2_I_9_ has a stronger attenuation than other perovskites at the same thickness. The vertically structured X‐ray detector based on the high‐quality Cs_3_Bi_2_I_9_ single crystals possesses a *µτ* value of 7.97 × 10^−4^ cm^2^ V^−1^, which is much higher than that of the melt‐grown Cs_3_Bi_2_I_9_ crystals. The sensitivity is 1652.3 µC Gy_air_^−1^ cm^−2^ at 50 V mm^−1^, as shown in **Figure** **15**a, more than 3.7 times higher than that of *α*‐Se detectors. And as depicted in Figure 15b, the SNR is calculated as 6.8 when the device was exposed under the dose rate of 130 nGy_air_ s^−1^. Zhang et al. also reported a low‐cost top‐seed solution (TSS) method with a constant growth rate, simple system, and high yield to grow high‐quality Cs_3_Bi_2_I_9_ single crystals.^[^ ^33^ ^]^ The crystals synthesized by this method exhibit a much lower trap density of 2.56 × 10^9^ cm^−3^, a high resistivity of 3.88 × 10^12^ Ω cm, and a higher *µτ* value of 1.35 × 10^−3^ cm^2^ V^−1^. As shown in Figure 15c, the X‐ray detector based on Au/Cs_3_Bi_2_I_9_ single crystal/Au exhibits a sensitivity of 4382 µC Gy_air_^−1^ cm^−2^ under the bias voltage of 20 V mm^−1^. The calculated SNR reaches up to 27.9 at the dose rate of 7.93 nGy_air_ s^−1^, which demonstrates the LoD is obviously lower than that of the X‐ray detector based on Cs_3_Bi_2_I_9_ prepared by the ITC method, as shown in Figure 15d. Except for Cs_3_Bi_2_I_9_, Li et al. introduced X‐ray detectors based on Cs_3_Bi_2_Br_9_.^[^ ^70^ ^]^ The Cs_3_Bi_2_Br_9_ single crystal with a 2D layer structure is prepared via the modified vertical Bridgman method. The high resistivity, transmittance, and bandgap of 6.8 × 10^11^ Ω cm, 80%, and 2.57 eV, respectively, and trap density of 9.7 × 10^10^ cm^−3^ demonstrate the excellent potential of Cs_3_Bi_2_Br_9_ for X‐ray detection (Figure 15e). As depicted in Figure 15f, the device shows a sensitivity of ≈230.4 µC Gy_air_^−1^ cm^−2^ and a low and no‐drift dark current density of 17.8 pA mm^−2^, and the 1 mm thick Cs_3_Bi_2_Br_9_ single crystal possesses an attenuation coefficient of 98.1%, compared with CdTe (99.4%), MAPbBr_3_ (90.6%), *α*‐Se (87.0%), and Si (10.6%). In addition, changing the type of A‐site cation will also change the structure of the lattice, such as (NH_4_)_3_Bi_2_I_9_ and Rb_3_Bi_2_I_9_ with the 2D structure are different from Cs_3_Bi_2_I_9_ and (MA)_3_Bi_2_I_9_ with the 0D structure.

**Figure 16:**
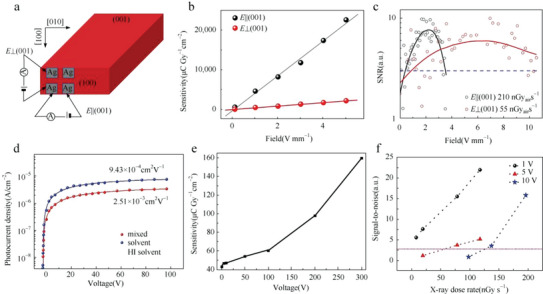
a) A planar‐type photodetector device made on the (100) surface of (NH_4_)_3_Bi_2_I_9_ single crystal. b) X‐ray sensitivities and c) signal‐to‐noise ratio (SNR) of the devices in directions parallel and perpendicular to the (001) surface. Reproduced with permission.^[^ ^57^ ^]^ Copyright 2019, Springer Nature. The blue dotted line represents an SNR of 3, so the detection limits are 210 nGy_air_ s^−1^ for parallel and 55 nGy_air_ s^−1^ for perpendicular devices, respectively. d) Voltage‐dependent photoconductivity of Rb_3_Bi_2_I_9_ single crystal produced by mixed solution and HI solution. e) X‐ray sensitivity of the optimized Rb_3_Bi_2_I_9_ single crystal under different voltage. f) Signal‐to‐noise ratio of the device. The dashed line represents the signal‐to‐noise ratio as 3. Reproduced with permission.^[^ ^111^ ^]^ Copyright 2020, WILEY‐VCH Verlag GmbH & Co. KGaA.

Zhuang et al. reported an X‐ray detector based on (NH_4_)_3_Bi_2_I_9_ single crystal with a 2D layered structure.^[^ ^57^ ^]^ The crystal possesses anisotropic electronic properties, and in order to measure its ability to detect X‐rays, the device is designed with the structure as shown in **Figure** **16**a. Due to its high average atomic number, 0.99 mm thickness of (NH_4_)_3_Bi_2_I_9_ is enough to attenuate 99% of the incident X‐ray photons, which demonstrates a much higher attenuation efficiency than Si and MAPbBr_3_. And being comparable to the MAPbBr_3_ and CdZnTe single crystals, (NH_4_)_3_Bi_2_I_9_ has *µτ* products of 1.1 × 10^−2^ and 4.0 × 10^−3^ cm^2^ V^−1^ in the parallel and perpendicular directions, respectively. And the parallel direction device achieved a large sensitivity of 8 × 10^3^ µC Gy_air_^−1^ cm^−2^, as shown in Figure 16b, which is comparable to MAPbBr_3_ and MAPbI_3_, while the perpendicular device exhibits a low detection limit as the maximum value of SNR is much larger than the parallel direction device shown in Figure 16c. The results demonstrate the potential of (NH_4_)_3_Bi_2_I_9_ in the anisotropic X‐ray detection performance, owing to the anisotropic electronic properties. Xia et al. also introduced Rb_3_Bi_2_I_9_ with the same 2D layered structure and proposed the structural restriction for the formation of 2D A_3_B_2_X_9_ perovskites, which is directly related to the value of octahedral factor *µ*, tolerance factor *t*, and the ionic radii of *A*.^[^ ^111^ ^]^ To optimize the crystal growth process, a mixed solution of HI and deionized water is used to replace the HI solution, and Figure 16d shows enhancement of the *µτ* product of the device, which increases from 9.43 × 10^−4^ to 2.51 × 10^−3^ cm^2^ V^−1^. Benefitting from this, the sensitivity of the device is calculated to be 42.5 µC Gy_air_^−1^ cm^−2^ under an electric field of 1 V mm^−1^ and can reach up to 159.7 µC Gy_air_^−1^ cm^−2^ with increasing the bias to 300 V mm^−1^, as shown in Figure 16e. And the SNR under different bias voltages under a range of X‐ray dose rates shown in Figure 16f demonstrates a low detection limit of 8.32 nGy_air_ s^−1^ under an electric field of 1 V mm^−1^ because the noise current increases with the increase in bias voltage.

**Figure 17:**
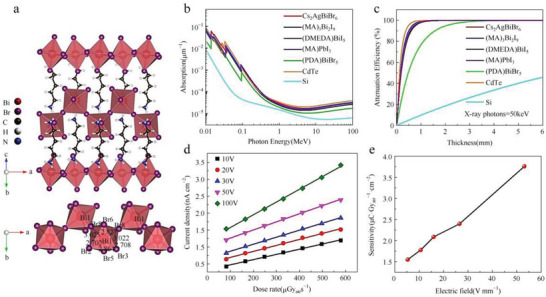
a) Perspective view of the (PDA)BiBr_5_ structure and zigzag chain of inorganic BiBr_6_ octahedra. b) The attenuation coefficient of CdTe, Si, MAPbI_3_, Cs_2_AgBiBr_6_, (NH4)_3_Bi_2_I_9_ and (DMEDA)BiI_5_ and (PDA)BiBr_5_ as a function of photon energy. c) The attenuation efficiency of CdTe, Si, MAPbI_3_, Cs_2_AgBiBr_6_, (NH_4_)_3_Bi_2_I_9_ and (DMEDA)BiI_5_ and (PDA)BiBr_5_ to 50 keV X‐ray photon energy versus thickness. d) The response current density as a function of the dose rate for various bias voltages. e) Device sensitivity under different electric fields. Reproduced with permission.^[^ ^119^ ^]^ Copyright 2021, Wiley‐VCH GmbH.

By engineering the composition of Bi‐based perovskite variants to change the conformation, the emerged (H_2_MDAP)BiI_5_ and (DMEDA)BiI_5_ perovskites with 1D structure, where the BiI_6_ octahedra are connected in a zigzag fashion, exhibited potential applications in direct X‐ray detection.^[^ ^117^, ^118^ ^]^ Likewise, Xu et al. reported a Bi‐based perovskite variant of (PDA)BiBr_5_ (PDA = pentamethylenediamine) with a similar structure to the reported (H_2_MDAP)BiI_5_, as depicted in **Figure** **17**a.^[^ ^119^ ^]^ The 1D zigzag chains of corner‐shared distorted BiBr_6_ octahedrons are interleaved by organic PDA cations. The crystal prepared via the antisolvent‐assisted crystallization method using CH_2_Cl_2_ as the antisolvent has a low density of defects of 2.0 × 10^10^ cm^−3^ and a direct bandgap of 2.71 eV determined by the BiBr_6_ network. The device based on Au/(PDA)BiBr_5_/Au exhibits a relatively high resistivity of 2.13 × 10^11^ Ω cm leading to a low dark current and a decreased noise current. The photon energy‐dependent X‐ray absorption coefficient and the thickness‐dependent attenuation efficiency (for 50 keV photons) are shown in Figure 17b,c. The dose rates‐dependent response current to X‐ray irradiation under different bias voltage is calculated in Figure 17d. The vertical (PDA)BiBr_5_ detector shows a sensitivity of 3.8 µC Gy_air_^−1^ cm^−2^ at 100 V (Figure 17e), demonstrating an inferior sensitivity compared to the Pb‐based perovskite.

**Figure 18:**
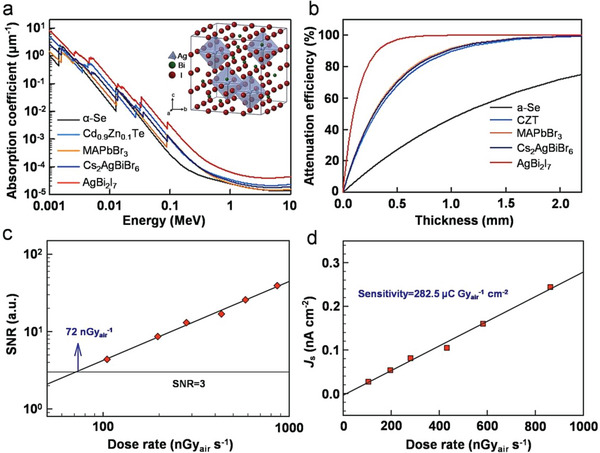
a) Absorption coefficient of different semiconductors. Inset: crystal structure of AgBi_2_I_7_. b) Attenuation efficiency versus thickness of semiconductors at 100 keV X‐ray photons. c) X‐ray dose rate‐dependent signal‐to‐noise ratio (SNR) of the device. d) X‐ray dose rate‐dependent signal current density (*J*_s_) of the device. Reproduced with permission.^[^ ^112^ ^]^ Copyright 2020, American Chemical Society.

Besides, Tie et al. synthesized AgBi_2_I_7_ with 0D structure by the vertical Bridgman technique with a bandgap of 1.73 eV, where each Ag^+^ is coordinated with six I^−^ to form an octahedron, while each Bi^3+^ coordinates with eight I^−^ to form a hexahedron, and BiI_3_ hexahedra and AgI octahedra are connected by corner‐sharing in 3D space, as depicted in the inset of **Figure** **18**a.^[^ ^112^ ^]^ The absorption coefficient and attenuation efficiency curves are shown in Figure 18a,b. The results demonstrate that AgBi_2_I_7_ exhibits a higher absorption coefficient and attenuation efficiency, and about 97% of the incident X‐rays can be absorbed by a 0.5 mm thick AgBi_2_I_7_. In addition, the device with the structure of Au/AgBi_2_I_7_/Au shows excellent environmental, thermal, and radiational stability, a low detection limit of 72 nGy_air_ s^−1^ (Figure 18c), and a high sensitivity of 282.5 µC Gy_air_^−1^ cm^−2^ (Figure 18d) due to the low dark carrier concentration, relatively high resistivity (1.3 × 10^8^ Ω cm), and balanced mobility (672.2 cm^2^ V^−1^ s^−1^) of AgBi_2_I_7_.

### 4.3 Te/Sb‐Based Perovskite X‐Ray Detectors

In addition to the Bi‐based perovskites mentioned above, Cs_2_TeI_6_ perovskite with a high resistivity is also widely used in X‐ray detectors.^[^ ^120^, ^121^, ^122^ ^]^ Cs_2_TeI_6_ is a deficient perovskite with a 0D structure and a band gap of ≈1.58 eV. The stopping power illuminated by 50 keV X‐ray photons of Cs_2_TeI_6_ is close to that of CdTe. Xu et al. reported a multilayer X‐ray detector based on Cs_2_TeI_6_ thick films.^[^ ^123^ ^]^ Benefitting from these and its high atomic number of elements, high electrical resistance, and high air and moisture stability, the device based on Cs_2_TeI_6_ films exhibits a high resistivity of 4.2 × 10^10^ Ω cm and good linear behavior even under the electrical field strength of 4000 V cm^−1^. The X‐ray response of Cs_2_TeI_6_ thin‐film devices under cathode irradiation at room temperature was studied by using weak silver X‐ray radiation. The device showed a linear response both in the dark and under 40 kVp, 10 µA, and 40 µA irradiation. The sensitivity is 19.2 µC Gy_air_^−1^ cm^−2^ under 40 kV_p_ X‐rays at an electrical field of 250 V cm^−1^, which is about 20 times higher than that of the hybrid 3D perovskite polycrystalline film X‐ray detector and comparable to those of established flat panel X‐ray detectors. The performance of the device can also be further enhanced by optimizing the Cs_2_TeI_6_ crystal quality to reduce carrier trapping.

**Figure 19:**
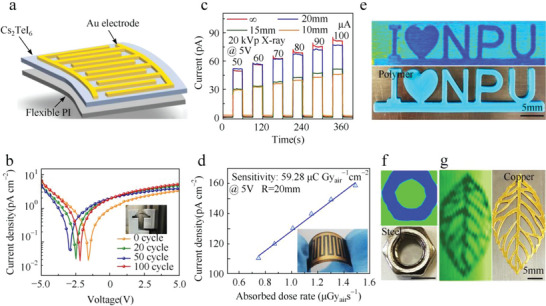
a) The flexible Cs_2_TeI_6_ detector structures. b) The *J*–*V* curves of the pristine detector for bending cycles up to 100. c) *I*–*T* response of the flexible Cs_2_TeI_6_ detector in bending state. d) The current density as a function of the dose rate at the bending radius of 20 mm. e–g) X‐ray images of polymer letter pattern, standard M6 nut, and electroplated copper leaf. Reproduced with permission.^[^ ^124^ ^]^ Copyright 2021, American Chemical Society.

Afterward, flexible X‐ray detectors based on Cs_2_TeI_6_ films are developed by Xu's group.^[^ ^124^ ^]^ Interdigitated Au electrodes with a thickness of ≈100 nm and electrode width and gaps of 0.5 mm were evaporated on the Cs_2_TeI_6_ films and the device structure is shown in **Figure** **19**a. To verify the stability after bending, the *J*–*V* curves are measured under different bending cycles (bending radius = 10 mm). No obvious deterioration in the resistivity of the flexible Cs_2_TeI_6_ film is observed, indicating that cracks did not occur in the film (Figure 19b). Additionally, the Cs_2_TeI_6_ detector exhibits periodic response under the bending radii (*R* = *∞*, 20, 15, and 10 mm) at 5 V bias. The sharp decline of photocurrent in a smaller bending radius is due to the decrease in the X‐ray dose rate under bending conditions (Figure 19c). Even if the X‐ray is not completely attenuated, the detection sensitivity of the flexible detector can still reach 59.28 µC Gy_air_^−1^ cm^−2^ at the bending radius of 20 mm (Figure 19d). The flexible Cs_2_TeI_6_ X‐ray detector demonstrates a good recognition ability under a low‐dose rate (59.21 µGy_air_ s^−1^). Three materials (polymer, steel, and copper) are imaged by using the X‐ray detector, as seen in Figure 19e–g. Cation engineering can induce dimensional evolution of halide perovskites. Centimeter‐sized 2D 4‐fluorophenethylammonium antimony iodide (FPEA_3_SbI_6_) single‐crystal perovskite has been synthesized by Li et al.^[^ ^125^ ^]^ Considering the vacancies in the 2D FPEA_3_SbI_6_ perovskite, they proposed to alloy Sn^2+^ ions into the crystal lattice to eliminate vacancy defects. As‐prepared FPEA_3_SbSn*_x_* I_6+2_ *_x_* single‐crystal perovskite X‐ray detector exhibits high sensitivity of 3160 µC Gy_air_^−1^ cm^−2^ when the Sb^3+^/Sn^2+^ molar ratio is 2.03. As shown in **Table** **3**, the sensitivity of Te/Sb‐based X‐ray detectors is generally inferior to other types of perovskite X‐ray detection devices, mainly due to low absorption coefficient and low *µτ* products. However, the most exciting thing is that the Cs_2_TeI_6_ perovskite films exhibit excellent air and moisture stability after flashing by the running water.

**Table 3:** Device performance of Te/Sb‐based perovskite X‐ray detectors

Device structure	Electric field [V mm^−1^]	Sensitivity [µC Gy_air_^−1^ cm^−2^]	Detection limit [nGy_air_ s^−1^]	Ref.
Au/Cs_2_TeI_6_ single crystal/Au	2.76	27.8	72.5	[120]
Au/Cs_2_TeI_6_ film/Au	6.67 × 10^3^	226.8	115	[121]
Au/Cs_2_TeI_6_ film/Au	10	76.27	170	[124]
C_60_/BCP/Cr/FPEA_3_SbISn_0.5_I_7_/Au	327.86	3160	389	[125]

## 5 Challenges and Outlook

Although lead‐free perovskite has already shown huge advantages for X‐ray detection, there are still quite a few challenges to overcome, such as how to stabilize the material and interface electrical properties under large bias and how to increase its stability in the atmosphere.^[^ ^126^, ^127^, ^128^ ^]^ Many researchers have demonstrated that the performance of detectors can be improved by using optimized synthesis methods to reduce defects and interface states, enhance carrier transport characteristics, and so on. Meanwhile, due to X‐ray's strong penetration, thicker perovskite materials are required to achieve complete X‐ray blocking. Because the thick film can be coated on any substrate, the array electrode with high density can be prepared on the substrate in advance to realize the preparation of a large size and high integration detector array and achieve high‐resolution imaging.

The blade‐coating process encourages the large‐area preparation of mm‐thick perovskite films. However, it is difficult to control the crystallization process when the solvent slowly evaporates. To improve the crystalline quality, the isostatic pressing method can be used to prepare high‐quality perovskite thick films. Considering the pressure that applied to the film increases sharply when preparing large‐scale thick films, posing a significant challenge to hydraulic equipment. As an alternative, the melting and hot‐pressing method may be the most promising technology to prepare high‐quality and large‐size mm‐thick films. Although much progress has been made in the synthesis of perovskite single crystals, there are still difficulties in flip‐chipping and cutting in order to achieve integration with circuits due to their relatively poor mechanical properties. Therefore, direct integration on the substrate may be an alternative strategy.^[^ ^129^, ^130^ ^]^

To improve device stability, encapsulation is one of the most direct strategies for isolating oxygen, water, and other substances from the environment. The ionic migration in perovskite leads to dark current drift, which degrades the device performance. Growing high‐quality single crystals with adjusted compositions and surface/interfacial passivation can inhibit ion migration, thereby enhancing the sensitivity and stability. In addition, more stable inorganic perovskites, such as Cs_2_AgBiBr_6_, can be used for constructing high‐stable X‐ray detectors.

The correlations between the intrinsic properties of perovskite and the key figures‐of‐merit of the detectors have been partially revealed, which could provide valuable insights into the remaining bottlenecks for the detector development, such as large dark current, baseline drift, and so on.^[^ ^131^ ^]^ Many reported perovskites exhibit severe ion migration, resulting in baseline drift. And the problem causes deteriorated imaging resolution, reduces sensitivity, dulls response speed, accelerates the decomposition of perovskites, and corrodes metal electrodes, which decreases the stability and restricts the application in X‐ray detectors. Besides, the performance of direct radiation detectors is mainly focused on sensitivity and detection limit, and there are rare studies on their comprehensive performance such as response speed, spatial resolution, and dynamic characteristics. It is necessary to cooperate with commercial companies to prepare detection systems and study the overall performance of detectors.

The X‐ray detectors based on photon counting can detect each photon separately which makes it possible to count the X‐ray with a very wide energy spectrum by energy region and determine the energy range to which it belongs.^[^ ^132^, ^133^ ^]^ To obtain more information from X‐rays of different energies, the development of dual energy or multienergy resolution X‐ray detection is an important research direction in the future X‐ray imaging field.^[^ ^134^ ^]^ However, as detectors with high‐resolution requirements, X‐ray detectors based on photon counting face the following constraints: when the counting rate is high, different photons interact with substances to produce superposition, which greatly reduces the spatial resolution. Therefore, the future development direction is to improve the response speed of detectors and the processing speed of back‐end electronics.

## Conflict of Interest

The authors declare no conflict of interest.

## Author Contributions

X.G. and Y.‐A.C. drafted the manuscript. X.G., Y.‐Y.L., J.R., G.‐H.D., K.Q., Z.L., and J.P. discussed and revised the manuscript. H.T., Y.Y., D.X., and T.‐L.R. revised and finalized the manuscript.
